# Gender stereotypes and professional experiences of female nurses in Türkiye

**DOI:** 10.3389/fpubh.2025.1538517

**Published:** 2025-01-24

**Authors:** Zeynep Aca, Arzu Kırcal-Şahin, Akın Özdemir, Yavuz Selim Kaymakcı

**Affiliations:** ^1^Department of Social Work, Faculty of Health Sciences, Bandırma Onyedi Eylül University, Balıkesir, Türkiye; ^2^London School of Economics and Political Science, London, United Kingdom; ^3^Department of Labour Economics and Industrial Relations, Faculty of Political Sciences, Sakarya University, Sakarya, Türkiye; ^4^The Graduate School, Manisa Celal Bayar University, Manisa, Türkiye

**Keywords:** female nurses, gender inequities, healthcare inequality, professional growth, workplace discrimination

## Abstract

**Introduction:**

Gender roles and stereotypes play a significant role in shaping the nursing profession, perpetuating systemic inequities that negatively impact professional experiences and healthcare system efficiency. In Türkiye, patriarchal norms and systemic disparities exacerbate these workplace challenges, particularly for female nurses.

**Methods:**

This qualitative study utilized semi-structured interviews with 13 female nurses working in intensive care units to examine the influence of societal expectations, workplace discrimination, and institutional policies on gender inequities in nursing.

**Results:**

The findings reveal that cultural norms, family influence, and constrained career planning often channel women into nursing, reinforcing perceptions of the profession as an extension of caregiving roles. While participants rejected the notion of nursing as a “women’s profession,” their narratives highlighted the pervasive impact of gendered expectations. Additionally, political favoritism and nepotism were identified as factors exacerbating workplace challenges, reflecting broader systemic issues in Türkiye’s labor market. The normalization of gender norms and their internalization by female nurses further complicate efforts to combat discrimination.

**Discussion:**

The study underscores the necessity for policy interventions to address systemic gender inequities in nursing. Recommendations include implementing mandatory gender equality education within healthcare institutions, stricter enforcement of anti-violence laws, and the establishment of psychological and legal support systems for workplace violence victims. Additional measures, such as childcare support and regulations against marital status-based discrimination, are essential to mitigate inequities. By addressing societal, cultural, and institutional factors, this research provides actionable strategies for healthcare organizations and policymakers to promote equity and improve sector efficiency.

## 1 Introduction

Despite legal regulations in Türkiye, such as the principle of equal pay for equal work in labor law, inequalities between men and women persist in various spheres. For instance, although nursing is a predominantly female-dominated profession globally, the employment rate of women in Türkiye (30.4%) significantly lags men (65%) (1). Gender inequality is also reflected in the Gender Inequality Index, where Türkiye has ranked between 133rd and 127th out of 146 countries over the past 5 years (115, 116).

Healthcare is one of the most fundamental human rights. Every citizen is entitled to qualified, uninterrupted, and accessible healthcare, with nurses delivering approximately 80% of these services. Nurses play a pivotal role in providing human-centered, equitable, and high-quality care, which is critical for sustainable healthcare services. Investment in nursing yields substantial returns, including improvements in public health, shorter hospital stays, and reduced errors leading to patient harm. Research suggests a 1% increase in nursing intensity can raise life expectancy at birth by 0.02% (2).

The World Health Organization (WHO) defines health technology as methods, equipment, and techniques used alongside healthcare providers that significantly contribute to addressing health-related issues (3). In nursing, technological advancements such as patient lifting devices and robotic systems have reduced physical strain, enhancing workplace safety and efficiency (4).

Among OECD countries, Türkiye ranks lowest in the number of nurses per capita (5). This data highlights the urgent need to address a nursing shortage in Türkiye. Retaining experienced nurses is critical, and understanding their challenges is key to eliminating gender-based inequities (6).

For example, the infant mortality rate in Türkiye is 9.1 per 1,000 live births, compared to the OECD average of 3.6. Similarly, the maternal mortality rate in Türkiye is 11.6 per 100,000 live births, exceeding the OECD average of 10.8 (5). These statistics underscore persistent systemic challenges in achieving equitable healthcare.

Gender stereotypes, as cognitive frameworks shaping perceptions of men and women (7), reinforce systemic inequities in nursing. This study explores their role in workplace discrimination and professional barriers, drawing from Cottingham et al. (8) and Heilman (7) to understand their interplay with gender roles, which dictate behavioral expectations (9). The healthcare sector, predominantly staffed by women, exemplifies systemic gender inequalities, evident at both individual and institutional levels (10). Despite the critical role of healthcare in societal welfare, the workplace experiences of women healthcare workers are profoundly shaped by gender-based discrimination (11, 12). Globally, women constitute 67% of healthcare and caregiving workers (13). However, according to the International Labor Organization (14), women in healthcare are 30% less likely to be promoted compared to male colleagues and face a 24% average pay gap, even in similar positions. These statistics highlight how gender norms undervalue women’s contributions, perpetuating workplace inequalities (114). Leadership disparities further marginalize women’s roles, with women occupying only 25% of senior positions in healthcare (15).

Addressing gender stereotypes in nursing is vital for public health, given the global nursing shortage and its impact on healthcare systems (16). WHO estimates that women constitute 67% of the global health and social workforce but remain underrepresented in leadership, comprising only 25% of senior roles (17). These disparities, coupled with systemic inequities, undermine healthcare efficiency and workforce sustainability, particularly in countries like Türkiye.

Gender stereotypes establish a persistent hierarchy favoring male nurses, particularly in promotions and roles requiring physical strength, which marginalizes female professionals. Participants highlighted workplace discrimination, such as verbal abuse and systemic favoritism, as key factors reducing motivation and productivity. These findings underscore how entrenched stereotypes perpetuate inequalities in healthcare systems (10). This stark timeline underscores the slow pace of progress and the need for more inclusive policies. The nursing profession, where women make up approximately 90% of the workforce (18), serves as a crucial context for analyzing the impact of gender-based discrimination and stereotypes in workplaces.

While gender inequalities in healthcare are a global issue, Türkiye offers a compelling case study for examining their deep-rooted impacts. In 2022, the Turkish Ministry of Health reported 1,350,528 healthcare workers, including 243,565 nurses (5). Although detailed gender distribution data for nurses is unavailable, nursing in Türkiye, as in many countries, is a predominantly female profession due to its historical association with caregiving roles. Patriarchal structures in Türkiye reinforce traditional gender roles, perpetuating systemic workplace inequalities.

Türkiye’s cultural norms, shaped by secular and Islamic traditions, reinforce patriarchal values that dictate women’s roles in public and private spheres. These beliefs emphasize caregiving roles for women and limit their professional autonomy, contributing to systemic workplace inequalities (20, 21). These norms intersect with political structures to perpetuate gender inequalities, particularly in professions like nursing, which are traditionally seen as extensions of domestic caregiving roles. In patriarchal societies like Türkiye, gender inequalities are systematically reproduced through professional practices, reflecting the broader cultural norms and values (22). Understanding the experiences of women nurses in Türkiye provides critical insights into the intersection of gender inequalities and public health.

Existing studies primarily focus on Western contexts, where healthcare systems differ significantly in structure and societal norms (35). This study addresses this gap by examining how these unique dynamics amplify workplace discrimination (24), hinder professional growth, and impact healthcare efficiency in Türkiye. Such an analysis highlights the broader implications of gender disparities for individual professionals and the quality of healthcare services.

Transformations in healthcare have begun to take place in Türkiye under the influence of neoliberalism. Among these transformations, a pivotal development was the Health Transformation Program (HTP) initiated in 2003 (25). The HTP aimed to achieve financial sustainability, quality, efficiency, and accessibility in healthcare services (26). With the amendment to the Nursing Law in 2007, the legal pathway was opened for men to become nurses, marking a significant step toward preventing gender discrimination in nursing (27). In alignment with this regulation, the definition of the nursing profession was made gender neutral.

In an effort to deliver healthcare services more effectively and efficiently, certain administrative changes in nursing were also implemented. With the issuance of Decree Law No. 663 in 2011, the traditional role of the chief nurse was replaced by the General Directorate of Health Services. According to the 2019 Directive on the Working Procedures and Principles of the Provincial Organization of the Turkish Public Hospitals Institution, the responsibilities of health services directors include the planning, execution, monitoring, and evaluation of care services, as well as the procurement, security, preparation, cleaning, disinfection, and sterilization of medical supplies required for care services. Additionally, they are responsible for the education, development, assessment, and supervision of personnel providing healthcare services (28).

Despite efforts to enhance quality and some progress achieved, the Health Transformation Program has led to the evaluation of patients as customers and placed customer satisfaction as the highest priority (29). This, in turn, has become one of the primary factors contributing to violence in healthcare. One of the core objectives of the program was “Quality and Accreditation for Qualified and Effective Healthcare Services.” In alignment with this goal, efforts have been directed toward the establishment of the Turkish Healthcare Quality System, as outlined in the “Regulation on the Improvement and Evaluation of Healthcare Quality,” published on June 27, 2015, in Official Gazette No. 29399 (30).

Furthermore, political decisions, such as Türkiye’s withdrawal from the Istanbul Convention in 2021, have exacerbated the visibility of gender-based rights violations across social, economic, and professional domains.

Healthcare-related violence remains a pressing issue, disproportionately targeting women nurses, with 43.3% of recorded incidents in 2023 directed at them (31). This alarming statistic reveals the extent of violence faced by healthcare workers, particularly women. Such experiences threaten psychological health, job satisfaction, and professional resilience, negatively affecting individual nurses and the systemic efficiency of healthcare services. The prevalence of violence and discrimination against women nurses underscores the need for a deeper examination of how gender roles and stereotypes shape their professional lives.

This study aims to investigate how gender roles and stereotypes affect the professional experiences of women nurses in Türkiye. Gender inequalities in healthcare not only undermine the professional well-being of women nurses but also diminish the overall quality of healthcare services (32). How are the professional experiences of female nurses in Türkiye framed by gender stereotypes? By addressing this research question, the study seeks to explore how individual discriminatory practices and institutional policies impact these professionals.

Inequality refers to measurable differences between groups, such as disparities in income, access to resources, or representation in leadership. Inequity, on the other hand, emphasizes unfairness or injustice in the distribution of opportunities or treatment. While inequalities may result from natural or structural differences, inequities highlight systemic biases or discriminatory practices that perpetuate unequal outcomes (Sen et al., 2007) (33).

Gender inequalities in the Turkish healthcare sector manifest in various ways. Women nurses are often viewed as extensions of caregiving roles, limiting their professional opportunities and creating significant barriers to leadership. These perceptions stem from societal norms that confine women to specific roles, which are continuously reproduced through organizational practices. At an individual level, women nurses face challenges such as discrimination, violence, and work-life balance issues. Systemically, gender norms influence organizational structures, creating barriers that undermine institutional equity and efficiency.

The literature extensively examines the impact of gender stereotypes on the nursing profession. For instance, the disproportionate ease with which men access leadership positions and the frequent marginalization of women’s professional contributions are identified as core dynamics of gender inequities within the healthcare sector (11, 12, 34, 35). However, limited attention has been given to the intersection of Türkiye’s religious, cultural, and political structures with gender stereotypes. This study addresses this gap by examining how these dynamics influence individual experiences and institutional practices in the Turkish context.

Existing studies in Türkiye primarily focus on topics such as male nursing students’ perceptions of the profession (36), societal views of male nurses (37), and the professional perspectives of final-year nursing students (38). While these studies provide valuable insights, they often overlook the specific challenges faced by women nurses. Research on the relationship between gender and nursing (39) has yet to comprehensively explore how stereotypes shape women’s professional experiences. Addressing this gap is essential for understanding the broader implications of gender inequalities in healthcare and formulating effective policies.

This study contributes to the literature in three key ways. First, it evaluates the individual and systemic effects of gender stereotypes within a socio-cultural and political framework, addressing gaps in international literature. By focusing on Türkiye, where traditional norms and patriarchal structures prevail, the study provides a broader perspective on gender dynamics in nursing. Second, it offers guidance for developing policies that promote gender equality in healthcare, using insights from women nurses’ experiences to inform both individual-level interventions and systemic reforms. Finally, the study highlights how societal norms shape the conflict between familial roles and professional responsibilities, providing a nuanced understanding of the impact of gender inequality on healthcare efficiency. These contributions fill a critical gap in academic literature and offer practical solutions for fostering equity in the healthcare sector.

## 2 Reasons why women choose nursing as a profession

Florence Nightingale, a pioneer of the nursing profession, argued that nursing was more suitable for women, reinforcing traditional gender roles and stereotypes. Feminist theories have critiqued male-dominated interpretations while highlighting how professions like nursing have historically been associated with “feminine” characteristics, arguing that these perceptions stem from gendered dynamics in society (40, 41). Studies consistently show that nursing is widely perceived as a female-dominated profession (16, 42, 43). Historical, theoretical, and empirical evidence underscores the influence of gender roles and stereotypes in shaping societal perceptions of professions and reinforcing the association between professions and gender. However, these perceptions are often influenced by masculine frameworks (7, 44–47). Nonetheless, the significant representation of women in nursing has disrupted patriarchal norms, reshaping the gender-profession dynamic in favor of women.

From a young age, societal norms encourage girls to pursue professions emphasizing traits such as compassion and caregiving, while boys are guided toward roles requiring strength and composure (48). These early influences transcend personal preferences and become embedded as social norms that play a critical role in shaping career aspirations. Consequently, career choices are influenced not only by individual interests and abilities but also by societal expectations (34, 49). Through socialization, individuals internalize these norms, often reinforced by role models, aligning their aspirations with what is deemed “normal” (47). Women, commonly associated with qualities such as kindness, empathy, and helpfulness, find nursing to be a profession that aligns with these socially prescribed traits (50). This association perpetuates the perception of nursing as a “natural” choice for women, deeply rooted in caregiving roles and societal expectations.

Historically, most cultures have linked women’s roles to biological reproduction and domestic responsibilities, such as childcare, meal preparation, and caregiving (11, 12, 16, 35, 51). These traditional roles have constrained women’s career opportunities, subjecting them to societal and cultural pressures that hinder strategic career planning and perpetuate challenges to achieving gender equality. Families often steer daughters toward roles perceived as compatible with caregiving or family values, while encouraging sons to pursue high-status, financially lucrative careers. Professions such as nursing, viewed as aligned with family values, further entrench gender roles and stereotypes, transforming them into societal norms (34, 49, 52, 53). This familial influence highlights the pivotal role of social norms in shaping career preferences and perpetuating gendered perceptions of professions. Conversely, men’s reluctance to pursue nursing stems from stereotypes associating the profession with low social status (52).

Gender theory posits that professions dominated by women are shaped by gender stereotypes. This perspective explains the persistent dominance of women in fields such as nursing, where gendered perceptions of caregiving and emotional labor prevail (35, 41, 42, 54). Emotional labor, a critical component of nursing, prioritizes emotion, attention, and care over task orientation in service delivery, reinforcing the association of the profession with feminine traits (52, 55). Consequently, women often emphasize their motivation to contribute socially when selecting careers, demonstrating how professions like nursing are shaped not only by individual preferences but also by societal expectations.

## 3 Gender-based professional discrimination in nursing

Gender plays a pivotal role in the division of labor in healthcare, serving as a key source of workplace discrimination (56). Research shows that gender influences the distribution of power and privilege in the labor market, perpetuating inequalities in employment (57). The gendered division of labor in healthcare, rooted in societal norms, continues to be a focal point of academic discussion. Perceptions of women feed into biases that sustain workplace inequalities (58). Gender discrimination, deeply embedded in socially constructed roles and norms, encompasses practices that hinder individuals’ full enjoyment of their rights. These norms not only underpin workplace inequalities but are also perpetuated through societal interactions, reinforcing structural barriers for women (51, 59).

Nursing is a profession shaped by patriarchal views that often marginalize women as a disadvantaged group (60). Patriarchy, as a cultural system, regards men as superior and perpetuates this dominance through oppressive gender roles and norms (61). In response, disadvantaged groups often adopt characteristics of dominant groups to gain power (62). This dynamic can lead female nurses to perpetuate discrimination against their peers by reinforcing gender stereotypes. Conversely, attitudes toward male nurses tend to be more flexible, favoring men and enhancing their authority within the profession (46, 51).

The phenomenological study by Salvador and Mohammed Alanazi (63) provides a comprehensive analysis of the challenges faced by male nurses in Saudi Arabia, highlighting the pervasive gender stereotypes, their resilience in overcoming workplace discrimination, and their vital contributions to advancing the Saudi Vision 2030 in healthcare. Gender-based discrimination in healthcare assigns women a secondary status, overshadowing their professional contributions.

The integrative review by Gauci (51) highlights persistent workplace gender discrimination within the nursing workforce, underscoring its detrimental effects on professional advancement, job satisfaction, and retention, and calling for comprehensive strategies to foster equity and inclusivity.

In caregiving roles like nursing, female nurses are often deemed less competent than their male counterparts and excluded from decision-making processes (46, 64). This perception deepens gender inequalities within the profession, diminishing women’s visibility and professional value. Workplaces characterized by pervasive gender discrimination reinforce women’s subordinate status while promoting male privilege through the “glass escalator” effect, where men experience accelerated career advancement (65, 66).

Female nurses are frequently undervalued and perceived as lacking specialization, which undermines their self-esteem and professional identity (54, 65). These discriminatory practices have far-reaching consequences, including reduced motivation, feelings of inadequacy, and lower quality of life (67). Gender discrimination normalizes differentiated roles deemed “natural” for women, entrenching societal acceptance of these inequalities and perpetuating systemic issues (47). This normalization makes it challenging for women to recognize workplace inequities and organize against them, further marginalizing gender-based issues (11).

Social structures heavily influence workplace dynamics, reinforcing gendered expectations that undermine women’s competence and subject them to degrading treatment, including harassment (16, 45, 61, 65, 66). Another manifestation of discrimination is the sexual objectification of female nurses, which associates the profession with feminine traits perceived as sexual symbols (68).

Workplace policies and practices are rarely gender-neutral (16). Men are often associated with competitiveness, while women are linked to altruistic, relational, and maternal roles (69). These dynamics affect perceptions of professional competence and status, hindering efforts to address workplace inequalities. Discussions of workplace gender discrimination frequently focus on factors such as motherhood, working hours, and work-life balance, which exacerbate barriers for women and reinforce sexist norms (11, 34, 70).

The nursing profession is deeply influenced by gender-based dynamics and discrimination. Female nurses face significant challenges in demonstrating their professional skills and advancing their careers due to pervasive stereotypes and structural inequalities. These barriers erode women’s confidence, visibility, and opportunities for professional growth, creating significant obstacles to achieving equality. Gender discrimination not only undermines women’s professional identities but also normalizes inequalities, perpetuating systemic injustices at both societal and professional levels.

## 4 Methodology

Qualitative research approaches, due to their exploratory nature, seek answers to research questions shaped by “how” and “what kind of” formulations [(71), pp. 107–109]. In this study, we aimed to answer the question, “How are the professional experiences of female nurses in Turkiye framed by gender stereotypes?” Consequently, we adopted a qualitative research approach, aligned with the structure of our research question. As Merriam [(72), pp. 13–19] states, studies conducted with a qualitative approach focus on meaning and the process of understanding rather than numerical data, with the researchers’ positions and subjective evaluations being central to this process. The research team’s expertise in disciplines such as social work, social policy, and labor economics, directly related to the topic, contributed to a multifaceted analysis of the dataset. Additionally, the presence of multiple researchers and the emphasis on gender equality among them facilitated the reshaping of subjective evaluations into a more neutral form through diverse perspectives during the empirical process.

The snowball technique involves contacting participants directly or indirectly connected and expanding the sample size based on their recommendations during field research (73). Initially, we conducted preliminary interviews with three female nurses to evaluate sample accessibility and the clarity of potential interview questions. Following the pilot studies, we conducted face-to-face interviews with 13 female nurses using the snowball sampling technique. During the interviews, the quality of the data obtained was prioritized over the number of participants. Specific data were sought from each participant, and the data collection process was concluded when participant responses began to repeat (72).

To ensure a saturated dataset, we required that the female nurses included in the sample have a minimum of 3 years of professional experience. The participants’ average experience was 3.6 years were based in 9 different intensive care units across various departments in public hospitals, including anesthesiology, surgery, cardiovascular, internal medicine, neonatal, pediatric, cardiology, pediatric emergency, and neurology. This diversity in workplace settings allowed for a nuanced understanding of the shared and unique challenges (74) faced by female nurses across departments. Their average age was 30 years; three participants had children, six were married, and seven were single.

In qualitative research, interview content takes a unique form shaped by the field. Semi-structured interviews consist of open-ended questions, with the number of questions subject to change during the research. Interviews progress naturally, with the researcher prioritizing the flow of the conversation over the order of the questions [(75), p. 191]. Our semi-structured interview questions sought answers to three main issues: why nurses choose this profession, the types of gender-based occupational discrimination they encountered in their professional lives, and how this discrimination affected their professional productivity. Additionally, participants’ remarks on patients’ and their relatives’ behaviors, cultural/political/religious aspects of Türkiye, and discrimination from female colleagues enriched the interview content. The adverse effects of occupational gender discrimination on work motivation, work-family balance, and hospital operations contributed to shaping our codes and categories during data analysis. We avoided using leading or compound questions in the interviews, as emphasized by Merriam [(72), p. 97].

**Figure 1:**
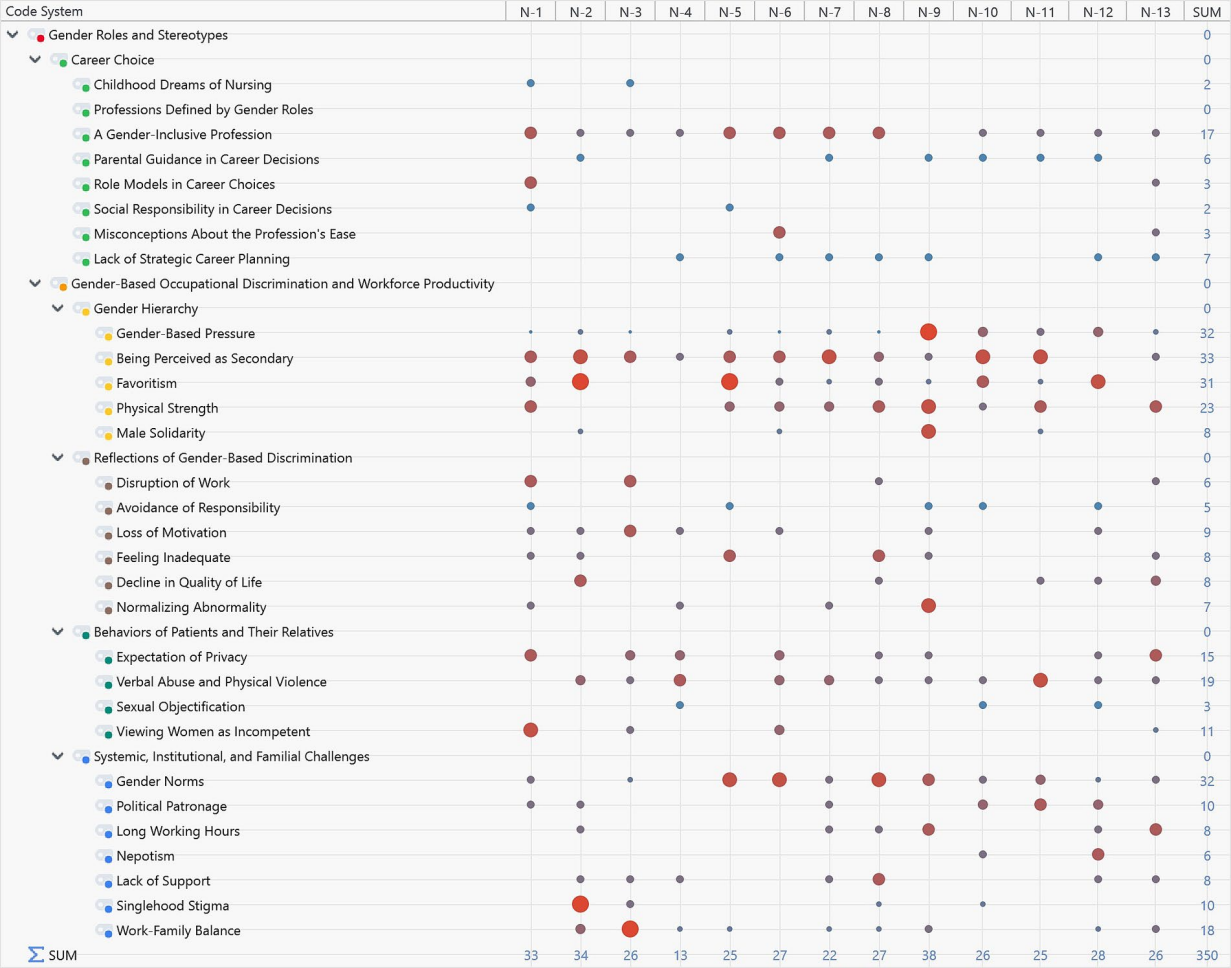
General view of the content analysis.

Content analysis is a technique that involves correlating the data obtained, highlighting emphases within expressions, and systematically classifying the data to present it in a more understandable and simplified form (76). Our content analysis revealed two categories explaining gender roles and stereotypes: “occupational choice” and “gender-based occupational discrimination and workforce productivity.” To elaborate on the latter, we identified four subcategories: “gender hierarchy,” “reflections of gender-based discrimination,” “patients’ and their relatives’ behaviors,” and “systemic/institutional and familial challenges.” As detailed under the Findings section (see Figure 1), these categories and subcategories were analyzed using 30 codes. In determining the codes, we adopted an inductive approach, primarily based on participants’ statements. Creswell [(75), p. 186] notes that an inductively shaped research process can adopt a deductive identity as findings are enriched by theory. Additionally, literature on the topic revealed studies corresponding to our categories and codes [e.g., (11, 12, 34, 35, 49, 51, 77)]. This allowed us to align our findings with existing literature through an inductive approach.

We followed a three-phase process for data coding: initial coding, focused coding, and theoretical coding [see (78, 79)]. During initial coding, researchers familiarized themselves with the dataset. Focused coding identified frequently emphasized statements, and theoretical coding examined the interactions between the code clusters we created. Although we predominantly used semantic coding during the coding process, we also employed emotion coding to understand participants’ emotional reactions, feelings, and states of mind. Semantic coding limits data interpretation to what participants explicitly stated or wrote, but supporting these codes with emotional expressions or psychological evaluations enhances the depth of content analysis (80, 81).

To resolve disagreements among researchers during the coding process, some authors [e.g., (82)] suggest numerical calculations. Peer review is also a frequently used method to ensure inter-coder agreement (72). Our research team reached a consensus on the final coding system through brainstorming at every stage of the three-phase coding process.

Computer-assisted qualitative data analysis programs provide researchers with significant advantages, particularly during the analysis of extensive datasets. These programs simplify findings with visual aids, making patterns more comprehensible [(75), pp. 195–196]. We used the MAXQDA qualitative analysis program during our content analyses.

Some methodologists suggest that the subjectivity inherent in qualitative approaches can transform into a more generalizable reality when the entire research process is transparently and thoroughly described. To mitigate the effects of subjectivity, expert review is considered an effective tool [(75), p. 202; (72), p. 221; (73), p. 288]. During the empirical process, including research design, code system creation, data analysis, analysis program usage, and reporting stages, we sought expert opinions from three academics experienced in qualitative research.

## 5 General view of the findings

This section presents the code-matrix browser generated using MAXQDA, offering an overview of the categories and codes derived from field research findings. The size of the circles in the matrix reflects the density distribution of participant statements within the coding system. For instance, statements by Participant N-2 predominantly cluster under the codes for favoritism and singlehood stigma.

Figure 1 provides an overview of the thematic codes and their frequency, highlighting key areas of participant concern such as ‘favoritism’ and ‘singlehood stigma’. Among these, the “career choice” category has no subcategories, while the second category, “gender-based occupational discrimination and workforce productivity,” is divided into four subcategories. A general review of the content analysis reveals that, with the exception of the “professions defined by gender” code, all codes are represented with varying levels of intensity.

The “career choice” category aims to uncover societal and individual dynamics influencing participants’ career decisions. The category “gender-based occupational discrimination and workforce productivity” seeks to identify the reasons for professional gender discrimination faced by nurses and its potential psychological and productivity-related consequences. Participants’ diverse characteristics—such as age, education, professional experience, marital status, and parenthood—contributed to the wide range of code densities seen in Figure 1, enriching the dataset.

## 6 Results

**Figure 2:**
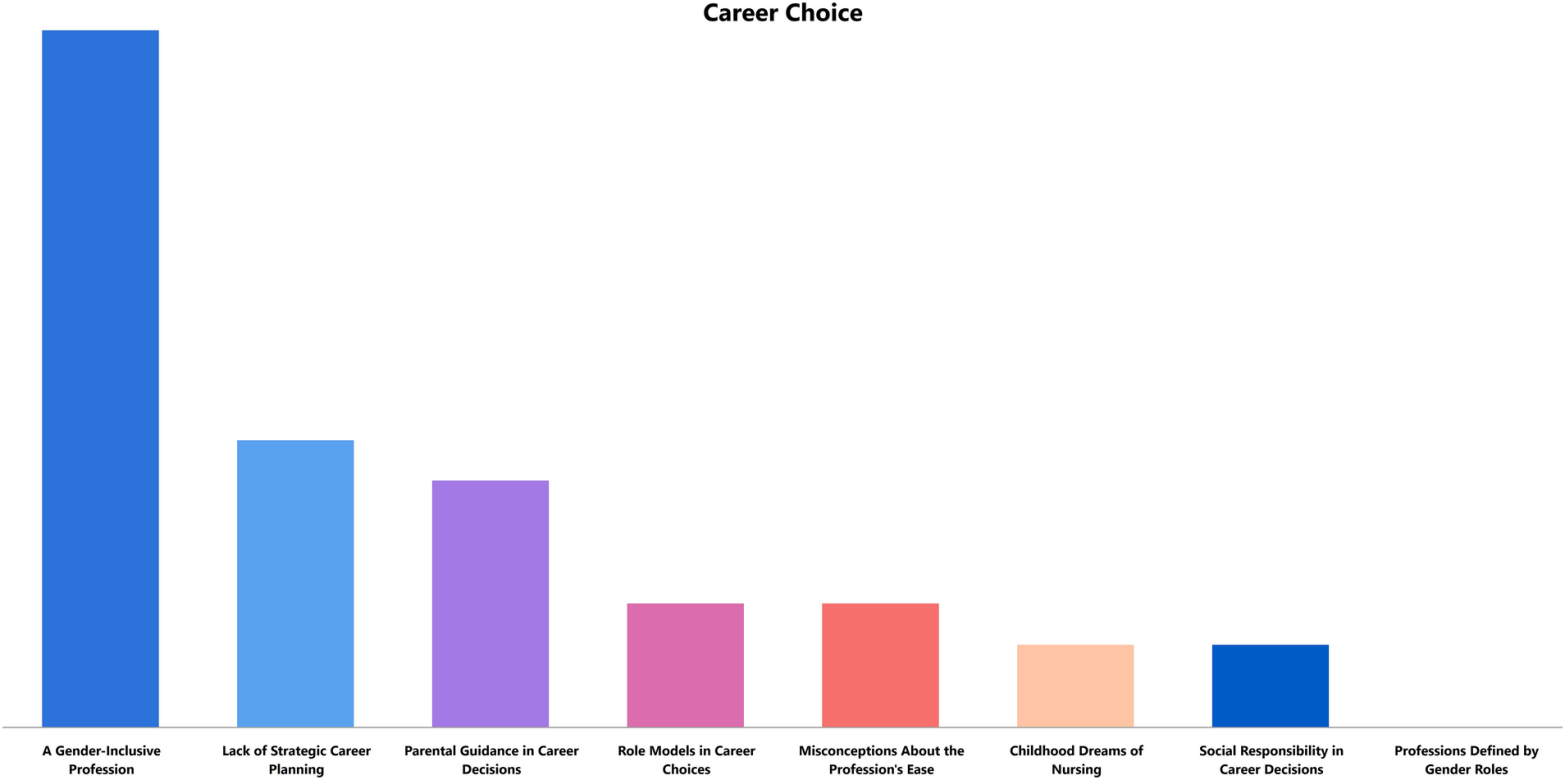
Career choice in nursing.

Figure 2 displays the distribution of codes regarding participants’ reasons for choosing nursing as a profession. Researchers categorized these reasons as childhood dreams, women’s profession, gender-neutral profession, family guidance, role modelling, social responsibility in career choice, misconceptions about the profession’s ease, and a lack of strategic career planning. Notably, the “professions defined by gender” code was the only one with no associated participant statements, suggesting that participants did not perceive nursing as exclusively a women’s profession. The predominance of the “gender-neutral profession” code further supports this finding. Supporting statements include:

“I’ve had many male nurse colleagues. I think men do this job quite well. It’s not correct to see nursing as only a women’s profession.”

“Nursing should not be classified by gender, just as psychologists are not labeled as male or female psychologists.”

A significant factor shaping career choice in nursing was a lack of strategic career planning. Inadequate guidance during pre-college education often led to an accumulation of statements under this code. Family influence also played a critical role in steering participants toward nursing careers. While participants generally rejected the notion of nursing as a women’s profession, they frequently mentioned parental encouragement rooted in gendered expectations during childhood. For example:

“My family wanted me to work in healthcare, believing that nursing was particularly suitable for a girl, so I did not want to disappoint them.”

Participants also noted that favorable working conditions in the public sector compared to the private sector led to a perception that nursing was an “easy” profession, a misconception they later recognized as unfounded. Some participants shared that they had aspired to become nurses since childhood and planned their careers accordingly. Additionally, participants often perceived nursing as a profession enabling women to contribute positively to society, as reflected in statements like:

“Nurses are incredibly compassionate people. That’s why I found myself more suited to this profession. I realized I wanted to work in healthcare to help others.”

**Figure 3:**
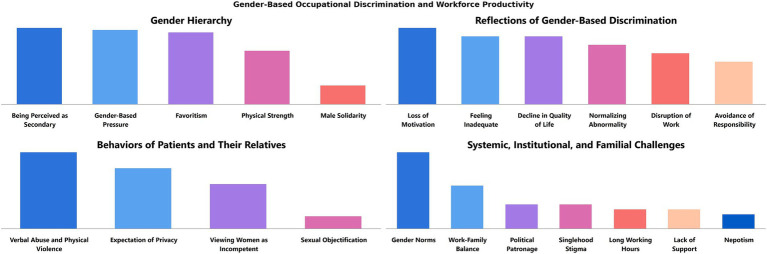
Gender-based occupational discrimination and workforce productivity in nursing.

Figure 3 highlights the societal, institutional, and familial causes of gender-based occupational discrimination experienced by participants and its impact on nursing productivity and nurses’ psychological well-being. A visible gender hierarchy, often favoring men, was frequently coded under the subcategory of “gender hierarchy.” This includes findings that women are seen as less valuable than men, their opinions are less respected, and they face greater occupational pressure simply for being women. Promotion processes also tend to favor men, reinforcing this hierarchy. However, some participants noted instances where women were preferentially treated in promotions:

“Women are given privileges in promotions, I’d say.”

Conversely, other participants viewed male nurses’ preferential treatment positively:

“Even our female supervisors say it’s good to have male nurses around. We prefer working with them, and their leadership creates a better work environment.”

While male nurses were occasionally perceived as discriminated against, the statements provided unique insights into the implicit dynamics of workplace discrimination, particularly from a female perspective:

“We work in intensive care, dealing with heavy patients [in terms of weight]. Male nurses are physically stronger when it comes to tasks like repositioning or lifting patients. I think we really need men in this profession.”

Statements like this underline how some female nurses, emphasizing physical strength, inadvertently reinforce the existing gender hierarchy.

### 6.1 Institutional and individual impacts of discrimination

Workplace gender discrimination manifests at both institutional and individual levels. Codes such as “work disruption” and “avoidance of responsibility” primarily highlight institutional impacts. Additionally, “loss of motivation” adversely affects both the individual’s psychological state and organizational dynamics. A participant’s statement captures these dual impacts:

“I do not want to go to work. I avoid shifts with someone who discriminates against me, which makes me tense and affects my job performance.”

The attitudes of patients and their families further exacerbate gender-based discrimination, diminishing workforce productivity. Participants reported experiencing verbal abuse and, in some cases, physical violence. Given the increasing incidents of violence against healthcare workers and women in Türkiye, these statements carry particular weight:

“I might not have faced professional discrimination, but I’ve encountered verbal abuse and physical violence.”

Sexual objectification, though less frequent, was also mentioned. This most egregious form of violence was deemed significant enough to warrant its own code. One participant shared:

“We experience verbal abuse, profanity, and harassment. Patients, even when fully conscious, approach us inappropriately.”

Participants also noted cultural dynamics in Türkiye, where Islamic conservatism influences patient preferences for male or female nurses, often tied to notions of privacy. A shortage of male nurses sometimes makes it difficult to meet these expectations. The following examples reflect such experiences:

“Male patients often prefer male nurses if they are conscious, and female patients insist on female nurses.”

“[Family members] think we are inexperienced or physically weak, saying, ‘You step aside, let someone else take over’.”

Cultural norms, workplace dynamics, and family expectations reinforce workplace gender discrimination. Gender roles and stereotypes, central to the content analysis, frequently became entrenched norms, affecting both male and female participants. For instance, long working hours and stigma against singlehood were found to reinforce gender norms across both genders. Male participants expressed that their perceived superiority in political and union relations exacerbated workplace discrimination:

“Men know how to leverage their political connections to get promoted. They are more active in unions, which allows them to work shorter hours and take fewer shifts.”

Additionally, nepotism emerged as a significant issue, with favoritism toward nurses married to doctors being a recurring theme:

“In our profession, those in better positions are often either well-connected in unions, politically influential, or married to doctors.”

The geographic and cultural context of Türkiye plays a crucial role in shaping workplace discrimination. Issues such as political favoritism, nepotism, and informal networks reflect the unique challenges of labor markets in countries where informal systems often take precedence over institutional structures.

## 7 Discussion and conclusions

This study analyzes findings under the main theme of “gender roles and stereotypes,” focusing on the categories of “career choice” and “gender-based occupational discrimination and workforce productivity.” These findings align with the gender inequality dynamics identified in the literature (11, 12, 16, 34, 35, 45, 51, 65, 66, 77) while also providing unique insights specific to Türkiye’s socio-cultural, religious, and political context. The structure of the healthcare sector, shaped by individual and systemic gender norms, reveals the multifaceted impacts of gender-based discrimination and stereotypes in the nursing profession.

**Figure 4:**
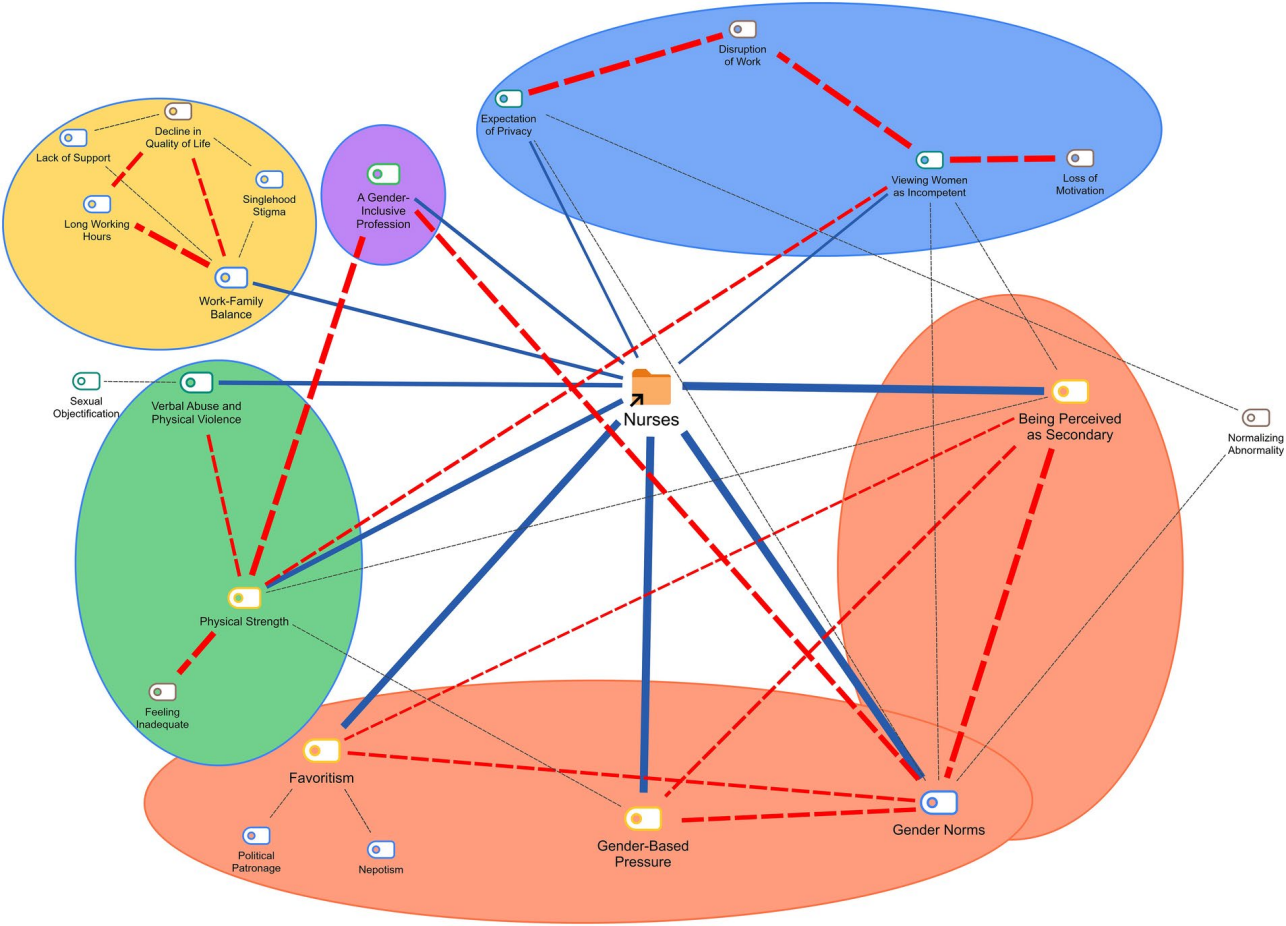
General view of all cases.

Figure 4 summarizes the patterns among the codes identified in all participant statements during content analysis. Starting with the “being perceived as secondary” code and moving clockwise, the blue lines represent the 10 most frequently mentioned codes, while red and black lines indicate co-occurring codes, with line thickness reflecting their intensity. The dashed red lines mark regions where co-occurring codes are concentrated. The colored circular areas highlight topics emphasized in the discussion and conclusions sections of our study. Below, we present the unique discussion points and conclusions derived from our findings under each category, comparing them with similarities or contrasts in the literature.

### 7.1 Career choice

Participants acknowledged the widespread societal perception of nursing as a “female profession.” However, their frequent assertion that nursing is a gender-neutral profession suggests their rejection of this societal norm. While this stance might seem like a challenge to gender norms, it implicitly underscores the persistent influence of these norms on career choices. The findings underscore the role of gender stereotypes in perpetuating systemic inequalities that contribute to nurse shortages and inefficient workforce utilization (11). Addressing these stereotypes is critical not only for fostering professional growth among female nurses but also for strengthening public health systems globally by ensuring equity in workforce distribution and leadership representation (83).

Family influence on career choices, persistent encouragement of daughters toward professions deemed feminine, and the predominance of female nurses as role models highlight the enduring impact of gender norms (34, 49, 52). Moreover, participants without strategic career plans were often directed by their families into this profession, inadvertently contributing to the entrenchment of gender roles and stereotypes.

Nursing, often associated with childhood aspirations, reflects how caregiving-oriented professions, influenced by gender roles, attract women from an early age (16, 35, 48, 50). This demonstrates that career choices are shaped not only by personal preferences but also by collective perceptions reinforced by societal norms and gender stereotypes (47).

The investigation by Gustafsson Sendén et al. (84) into dynamic gender stereotypes in Sweden revealed persistent perceptions of male agency and female communion, even in a highly egalitarian society, highlighting the influence of social roles on stereotype stability and the challenges of integrating communal traits into the male stereotype.

Globally, the perception of nursing as a gender-neutral profession is gaining traction in certain contexts. For instance, studies in Scandinavian countries and some parts of North America suggest that cultural shifts and policy interventions have contributed to reducing gender biases in nursing (85). However, these trends are unevenly distributed and are largely absent in regions where patriarchal norms and rigid gender roles dominate, such as in Türkiye. This contrast highlights the importance of socio-cultural contexts in shaping professional identities and opportunities.

### 7.2 Gender hierarchy

The codes under this category frequently intersected with one another and with gender norms (see Figure 4). Gender norms reinforce the subordinate status of female nurses while favoring male colleagues in leadership and promotions. Notably, some female leaders inadvertently perpetuate discrimination through stricter scrutiny of women, reflecting internalized biases shaped by patriarchal norms (51). This dynamic is compounded by the societal preference for male nurses, who are often viewed as more competent or suited to leadership roles (11, 35, 51, 77, 86, 87).

The term “secondary” in this context refers to the perception of female nurses as subordinate or less influential within healthcare hierarchies. This perception often comes from both institutional policies and societal norms, where nurses are viewed primarily as caregivers rather than decision-makers or leaders. For instance, physicians or administrative leaders may dominate decision-making processes, relegating nurses’ contributions to secondary importance. This status not only limits their professional agency but also undermines their visibility in shaping healthcare policies and practices.

An intriguing finding in this study is the role of female leaders in perpetuating gender-based discrimination, particularly through stricter enforcement of workplace rules and heightened scrutiny of female subordinates. This reflects an internalized bias rooted in patriarchal norms, wherein women leaders may subconsciously emulate or reinforce the very structures that have historically marginalized them. Understanding this dynamic is crucial for addressing workplace inequalities and promoting gender-sensitive leadership practices.

The findings align with global research on the stigmatization of nursing as a female profession and its impact on career progression. For example, in Saudi Arabia, male nurses have historically been integrated into the profession but still face stereotyping (63). What distinguishes this study is its focus on the interplay of Türkiye’s cultural, religious, and political structures with workplace dynamics, shedding light on how these factors uniquely amplify discrimination and inequities. By situating the findings within this specific socio-political context, the study contributes to the global discourse by highlighting region-specific barriers to gender equity in nursing.

In Türkiye, men were only officially admitted into the nursing profession in 2007. Despite this recent inclusion, male nurses have quickly ascended to leadership roles, often outpacing their female counterparts in promotions and organizational influence. This phenomenon highlights a broader systemic issue where patriarchal norms and gender stereotypes persist, even in professions traditionally dominated by women. Türkiye’s standing in the 2024 Global Gender Gap Report, ranked 127 out of 146 countries and the worst in Europe, underscores the entrenched nature of gender inequities that pervade professional domains, including healthcare.

Participants who felt undervalued regarding their professional skills indicated that nursing’s caregiving role is perceived as an extension of household responsibilities (46, 64, 112). Similarly, the perception of nursing as an auxiliary role to physicians contributes to the profession’s secondary status in workplace hierarchies, irrespective of gender (88). Some female nurses expressed the belief that managerial roles are better suited for men, citing qualities such as composure and problem-solving abilities, further entrenching gender norms (11, 12, 51, 52, 86).

The theoretical perspective presented by Xu et al. (89) highlights the significant role of transformational leadership in fostering nurses’ workplace social capital, emphasizing its potential to enhance relational networks, trust, and team cohesion, which are crucial for creating a healthy and productive work environment.

Such beliefs reflect an internalization of secondary status (90) and reinforce stereotypes (8).

The preference for men in leadership positions (8, 35, 54, 61, 65, 66, 69) perpetuates the bias that men are inherently more competent in authoritative roles (46). Interestingly, the numerical dominance of women in nursing sometimes results in preferential treatment for female nurses, highlighting the bidirectional nature of gender discrimination (113).

The most accepted hierarchical difference between men and women in nursing is physical strength. Frequently associated with men, physical strength is often the most internalized aspect of hierarchy by women (35, 51, 77, 86, 87, 91, 92).

The competences expected of nurses often encompass emotional labor, caregiving, and physical strength. These attributes, though central to nursing practice, are deeply influenced by gendered perceptions. Women are frequently associated with caregiving roles, while men are often perceived as better suited for leadership or physical tasks. Such stereotypes reinforce gendered divisions within the profession, impacting career trajectories and workplace dynamics.

Participants linked male nurses’ favoritism to this perceived need for physical strength, further supported by their relative scarcity in the profession (35, 47, 52).

### 7.3 Reflection of gender-based discrimination by patients and relatives

Participants frequently linked workplace discrimination with patient and family behaviors (see Figure 4), necessitating joint discussion of these categories. Women’s perceived lack of physical strength often leads to disrespect, verbal or physical abuse, and sexual harassment by patients or their relatives. These experiences diminish motivation and exacerbate feelings of inadequacy among female nurses (12, 16, 35, 45, 51, 54, 61, 65, 66).

In 2023, 43.3% of violence cases in Turkish healthcare targeted nurses, highlighting the risks associated with the profession (31). The “White Code” is an alarm system designed to ensure the swift response of security personnel to incidents where healthcare workers are subjected to violence, threats, or other criminal acts by patients or their relatives (93). This system was initiated by the Ministry of Health in 2015 with the aim of preventing violence against healthcare workers and ensuring their safety (94). The White Code can be activated through one of the following methods: Dialing 1,111 from any internal hospital line. Calling the 113 White Code Call Center. Filling out the form available at www.beyazkod.saglik.gov.tr (95). According to the Ministry of Health, intensive care units are among the locations where incidents of violence are most frequently reported. Notably, it is often not the patients themselves but their relatives who enter the intensive care units and perpetrate acts of violence (96).

Several countries have implemented successful interventions to address workplace violence in nursing:

- *The Netherlands:* The Buurtzorg model emphasizes team autonomy and supportive workplace environments, reducing burnout and conflict (97).
- *Australia:* Comprehensive workplace safety protocols, including mandatory reporting systems and staff training, have reduced violence incidents in hospitals (19).
- *Canada:* Zero-tolerance policies combined with robust legal protections for healthcare workers have improved workplace safety (13). Adopting similar interventions in Türkiye could mitigate the rising violence against nurses while fostering safer and more equitable work environments.

Studies show that female healthcare workers are often perceived as easier targets (11, 51, 68, 98). As a participant noted:

"We frequently encounter disputes with patients, such as questioning why they have to wait in triage. These questions are often harsher toward female nurses, sometimes escalating into insults, while they are addressed more amicably to male nurses."

The intimate nature of nursing often involves interactions with patients’ private spaces. In conservative societies, this can lead to preferences for same-gender caregivers, with a lack of male nurses intensifying challenges (35, 86, 91, 92). Participants reported that unmet privacy expectations can exacerbate nurse–patient issues, and women nurses are often forced to conform to societal norms at the expense of professional autonomy (see Figure 4).

### 7.4 Systemic, institutional, and familial challenges

As noted earlier, participants frequently linked gender norms with the codes in this category. Despite voicing concerns about these norms, participants often accepted them, normalizing what they perceived as abnormal over time.

The findings underscore the role of cultural and religious norms in shaping workplace discrimination. In Türkiye, cultural norms rooted in patriarchal values prioritize caregiving roles for women, marginalizing their professional contributions in leadership and decision-making. Religious conservatism, which often dictates gender-segregated spaces and privacy, further complicates gender dynamics, as female nurses are viewed primarily as caregivers rather than professionals (48, 98).

This process, observed both in our findings and the literature, highlights how individuals internalize gender differences in all social contexts, especially within professions (41–43). This finding aligns with Martin’s (99) assertion that people tend to internalize gender disparities across various domains.

This study reflects the unique dynamics of Türkiye’s labor market and healthcare sector. The workplace challenges identified in this study, such as verbal and physical harassment, lack of career progression, and gendered expectations, are amplified by Türkiye’s socio-cultural and political structures. These challenges, while not unique to Türkiye, are exacerbated in patriarchal and religiously conservative contexts. For example, studies in Saudi Arabia reveal similar dynamics, where male nurses face stereotyping and women remain underrepresented in leadership (63). The interplay of cultural, religious, and political factors in Türkiye creates a unique environment that amplifies these challenges in ways that are both locally specific and globally relevant.

The research by Barasteh et al. (100) identifies critical future challenges for the nursing system in Iran, emphasizing governance improvements, alignment of professional development with societal needs, and addressing human resource shortages to ensure sustainable nursing contributions to universal health coverage and public health.

The dominance of political patronage in employment relationships, particularly in the public sector, emerges as a critical finding, showcasing politically motivated favoritism irrespective of gender. The adaptation of the Buurtzorg model in Scotland through the INCA project demonstrated how self-managing healthcare teams can enhance care delivery by enabling staff autonomy to adjust care provision, which positively impacted patient outcomes.

Adopting elements of the Buurtzorg model in Türkiye could address systemic barriers by promoting nurse autonomy and integrating team-based care models. Key lessons include:

- Empowering nurses to take on leadership roles within care teams.
- Reducing hierarchical barriers between nurses and other healthcare professionals.
- Encouraging patient-centered practices that highlight the expertise of nurses. These strategies not only challenge gender stereotypes but also improve workforce efficiency and patient care quality, aligning with the goals of equitable healthcare systems.

Nevertheless, the findings underscored the necessity of establishing clear frameworks and fostering collaboration to address challenges related to team dynamics and management support (97).

Although union-related discrimination might seem to require a separate conceptual explanation, in Türkiye, unions—particularly in the public sector—often prioritize the political interests of their affiliates over members’ welfare. Therefore, union favoritism can be integrated into the broader framework of political patronage. Nepotism, arising from familial or political ties, is another significant form of favoritism observed in healthcare workplaces.

The underrepresentation of women in health leadership positions has broader implications for population health and national prosperity. Research shows that gender-balanced leadership leads to more inclusive health policies, which are critical for improving women’s and children’s access to healthcare services (19). For instance, female leaders are more likely to advocate for maternal health, family planning, and gender-sensitive healthcare practices. In their absence, systemic biases often perpetuate gaps in care, disproportionately affecting vulnerable populations and undermining public health outcomes. These inequities ultimately constrain national productivity and economic growth, as healthier populations are foundational to sustainable development (13).

The study by Khan et al. (101) underscores the significant economic costs of gender inequality in health and labor markets in India, emphasizing that improving women’s access to healthcare, education, and equitable employment opportunities could substantially enhance the country’s economic potential and sustainability.

The integration of artificial intelligence (AI) into nursing practices, as explored by Rony et al. (102), emphasizes the transformative potential of AI in enhancing preparedness and improving patient care outcomes within healthcare systems, highlighting its pivotal role in advancing nursing practice.

Long working hours, particularly in the context of extended shifts, negatively impact nurses’ work-life balance (11, 12, 34, 35, 51, 65, 67, 70).

While gender stereotypes and workplace discrimination reduce the efficiency of healthcare systems by impacting workforce motivation and retention, they also undermine the effectiveness of care delivery. Effectiveness in healthcare is closely tied to the well-being and professional growth of the workforce, as well as equitable opportunities for all genders. Addressing these issues aligns with the principles of value-based healthcare, which emphasize patient outcomes, workforce sustainability, and systemic equity. By fostering a more inclusive and equitable work environment, healthcare systems can enhance not only operational efficiency but also care quality and patient satisfaction.

The cross-sectional study by Werke and Weret (103) sheds light on the prevalence of occupational stress among nurses in public hospitals in Addis Ababa, Ethiopia, identifying significant factors such as rotating work shifts and parental responsibilities, which call for targeted interventions to reduce stress and improve healthcare service quality.

Despite these demanding schedules, female nurses disproportionately bear the burden of domestic responsibilities, which they reported as diminishing their quality of life (34, 58).

Gender stereotypes in healthcare contribute to significant productivity losses by reducing workforce motivation, increasing turnover rates, and perpetuating inefficiencies in task allocation. Studies indicate that workplaces with gender discrimination experience higher absenteeism and lower job satisfaction (104), which directly impact service delivery (10, 23). In Türkiye, these dynamics are further exacerbated by systemic biases that marginalize female nurses, leading to underutilization of their skills and limiting opportunities for career advancement.

The link between gender inequality and healthcare efficiency lies in workforce productivity and service delivery. Discriminatory practices such as unequal promotions, harassment, and overburdening female nurses with caregiving tasks reduce job satisfaction and retention, leading to workforce shortages. Studies have shown that equitable workplaces are associated with higher productivity and better patient outcomes (10, 23). In Türkiye, where gender norms further limit female nurses’ advancement, systemic inefficiencies arise from the underutilization of their skills and potential.

This study contributes to the literature on gender inequality in professional domains, particularly within healthcare systems. It builds on foundational works such as Heilman (7) on gender stereotypes, Rice and Barth (49) on gender roles, and recent studies like Gauci et al. (51) on workplace gender discrimination in nursing. By focusing on Türkiye, the study adds a socio-political dimension to the discourse, complementing global perspectives on the impact of cultural, religious, and institutional norms on gender inequities in healthcare.

The study’s second contribution lies in its exploration of the interplay between religion, cultural norms, and professional inequities. In Türkiye, religious conservatism often shapes gendered expectations in the workplace, reinforcing stereotypes about caregiving roles and influencing patient preferences for same-gender caregivers. These dynamics, while not unique to Türkiye, are exacerbated by the country’s socio-political context, providing valuable insights into the broader implications of religion in healthcare settings.

The persistence of gender stereotypes in nursing in Türkiye reflects broader societal influences rooted in cultural and religious norms. These norms not only reinforce traditional gender roles but also limit opportunities for women in leadership positions, perpetuating systemic inequities. Addressing these challenges requires comprehensive reforms that consider the socio-cultural and religious contexts, ensuring that policy interventions are both effective and culturally sensitive.

Another key issue is the role of marital status in deepening workplace discrimination. Single nurses (including married but childless nurses) often reported discrimination, particularly in shift and annual leave planning.

The recommendation to address marital status-based discrimination specifically refers to biases against unmarried and childless nurses. These individuals are often assigned less favorable shifts, overlooked for career advancements, or stigmatized as less committed to their roles compared to married or child-rearing colleagues. Addressing this issue requires clear institutional policies that ensure equitable treatment irrespective of marital or parental status.

Although this finding emerged from interviews with female nurses, it reflects a broader form of discrimination that can affect all nurses, regardless of gender.

Policy recommendations must address the deep-seated influence of cultural and religious norms. For example, public awareness campaigns should challenge traditional gender roles by promoting nursing as a gender-neutral profession. Mandatory gender equality education can also help reduce biases rooted in cultural and religious beliefs, fostering a more inclusive healthcare environment (23, 98).

## 8 Conclusion

This study provides a comprehensive analysis of how societal gender roles and stereotypes shape the professional and psychological experiences of female nurses in Türkiye. The findings align with similar studies in the literature while also revealing unique insights:

1. *Discrimination from Female Supervisors:* Female nurses frequently reported experiencing discrimination from female managers. This led some participants to advocate for increasing the number of male supervisors and nurses as a potential solution, diverging from prevailing literature on the issue.
2. *Resistance and Internalization:* While participants explicitly rejected societal gender norms, the factors influencing their career choices were heavily shaped by these norms. This paradox highlights how gender roles and stereotypes can subtly transform into accepted norms over time.
3. *Acceptance of Physical Limitations:* Some female nurses acknowledged the necessity of male colleagues for tasks requiring physical strength. This acceptance underscores the potential for discrimination complaints to evolve into tacit approval over time.
4. *Patient and Relative Behavior:* Female nurses were more likely to face verbal abuse, physical violence, or harassment due to perceived physical weakness. These incidents reflect a broader societal issue, with violence against women and healthcare workers (especially female nurses) on the rise in Türkiye.
5. *Non-Gender-Based Discrimination:* Discrimination driven by political, union-based, or nepotistic factors also exacerbates existing gender inequalities, highlighting the interconnected nature of systemic issues.
6. *Cultural and Religious Sensitivities:* Patients’ and relatives’ preferences for same-gender nurses, shaped by cultural and religious norms, disproportionately burden female nurses due to the limited number of male nurses in the profession.

### 8.1 Policy recommendations

To address these issues, we propose the following actionable policy recommendations for the healthcare sector in Türkiye:

1. *Mandatory Gender Equality Education:* Institutions providing healthcare training should offer mandatory courses on gender equality. These courses could help diminish stereotypes about women and encourage men to enter nursing with fewer prejudices.
2. *Stronger Enforcement of Anti-Violence Laws:* While laws exist to reduce violence against women and healthcare workers, their implementation faces significant challenges. Developing localized protocols tailored to regional and institutional needs could enhance the effectiveness of these regulations.
3. *Psychological and Legal Support Services:* Victims of workplace violence should have access to comprehensive psychological and legal support, institutionalized as a legal right.
4. *Childcare Support:* Childcare services and assistance should be provided to female healthcare workers, particularly those with demanding schedules. Addressing discrimination based on marital status through clear legal regulations is also essential.
5. *Public Awareness Campaigns:* Increasing the number of public service announcements addressing violence against female healthcare workers and scientifically evaluating the effectiveness of these campaigns could improve societal attitudes (105).

Policy recommendations must address the deep-seated influence of cultural and religious norms. For example, public awareness campaigns should challenge traditional gender roles by promoting nursing as a gender-neutral profession. Mandatory gender equality education can also help reduce biases rooted in cultural and religious beliefs, fostering a more inclusive healthcare environment (23, 98).

The recommendations are grounded in the study’s theoretical framework, which identifies gender stereotypes as a key driver of inequities at societal, institutional, and workplace levels. For instance:

- Mandatory gender equality education targets societal norms by challenging deeply ingrained stereotypes.
- Enforcement of anti-violence laws and support for victims addresses workplace-level discrimination and creates safer environments for nurses.
- Childcare support and marital status equity policies tackle institutional barriers that perpetuate systemic inequities.

By addressing these levels in a coordinated manner, the recommendations aim to dismantle the structural barriers sustained by gender stereotypes.

It is evident that achieving a healthy work environment, which forms the foundation of workforce efficiency and productivity, requires addressing gender inequalities in the workplace (89). Work environments where gender stereotypes cannot be transformed in favor of women are likely to have a negative impact on the efficiency and effectiveness of nursing professionals in the delivery of healthcare services.

The academic literature documents that improving gender-biased practices targeting nursing professionals positively influences every stage, from the quality of patient care to the delivery of healthcare services (89).

Retaining experienced nurses will further enhance the quality of care provided. For women, such initiatives may improve work-life balance and positively address the career-family dilemma faced by many women (89).

Implementing these recommendations requires leveraging existing structures within healthcare systems. For example:

- Hospital accreditations can include criteria on gender equity policies, mandatory training, and workplace safety, ensuring compliance with equity standards.
- Directors of nursing, as key decision-makers, can champion gender-sensitive practices by instituting fair scheduling, addressing discriminatory behaviors, and fostering inclusive leadership pipelines.
- In countries like the UK, where many hospital CEOs are qualified nurses, leadership has a critical role in driving systemic change. Similar strategies could be explored in Türkiye to promote leadership pathways for nurses, including women, to influence institutional practices from the top down. These systemic levers create accountability and sustainability for the proposed reforms.

In Türkiye, hospital leadership structures differ from countries like the UK, where many CEOs are qualified nurses (106). Instead, directors of nursing and administrative leadership often hold significant influence over policies affecting workforce dynamics. Empowering directors of nursing to advocate for gender equity and professional development can bridge this gap, ensuring that hospital-level reforms align with broader systemic goals (107). Future research could explore opportunities to elevate nursing professionals into executive roles, enhancing their capacity to drive systemic changes.

To ensure effective implementation of the proposed policies, healthcare institutions can:

- Integrate gender equity benchmarks into hospital accreditation standards, requiring periodic reviews and adherence to anti-discrimination policies (108).
- Establish training programs for hospital leaders, such as directors of nursing, to champion equitable practices and address systemic biases (109).
- Encourage partnerships with international organizations to adopt best practices for gender-sensitive healthcare environments (110).
- Develop local committees within hospitals to monitor and report on compliance with anti-violence laws and gender equity initiatives. These processes create accountability and embed equity into organizational practices, ensuring that policy recommendations translate into tangible outcomes (111).

These systemic levers create accountability and sustainability for the proposed reforms.

These strategies, tailored to the specific socio-cultural and political context of Türkiye, offer a pathway toward fostering equity in the nursing profession and the healthcare sector as a whole.

### 8.2 Limitations and directions for future research

The study’s primary limitation is its focus on Türkiye’s healthcare sector. While its findings on gender inequality may be broadly generalizable, cultural and political dynamics unique to other countries may lead to different outcomes. Additionally, the reliance on interviews primarily with intensive care unit nurses, due to snowball sampling, enriched the findings but also narrowed their scope. Expanding future studies to encompass all hospital units and including both male and female nurses could provide a more comprehensive understanding of the issue.

The emphasis on female participants highlighted the need to investigate discrimination faced by male nurses. Broadening the scope of research questions to include all genders would allow for a more balanced exploration of these dynamics. Additionally, adopting a gender-balanced research team in future studies could help minimize potential biases in data interpretation.

Finally, future research could involve comparative studies across different countries, sectors, and hospital units, as well as interdisciplinary collaborations supported by international funding. This study, despite its limitations, has provided valuable preliminary insights and serves as a foundation for broader scientific exploration.

## Data Availability

The original contributions presented in the study are included in the article/Supplementary material, further inquiries can be directed to the corresponding author.
