# Supplementary material for: Gender stereotypes and professional experiences of female nurses in Türkiye
Source: Front Public Health. 2025 Jan 24;13:1538517. doi: 10.3389/fpubh.2025.1538517 (PMC11803633; doi:10.3389/fpubh.2025.1538517)
Supplement: Supplementary file 1 [file Data_Sheet_1.PDF]

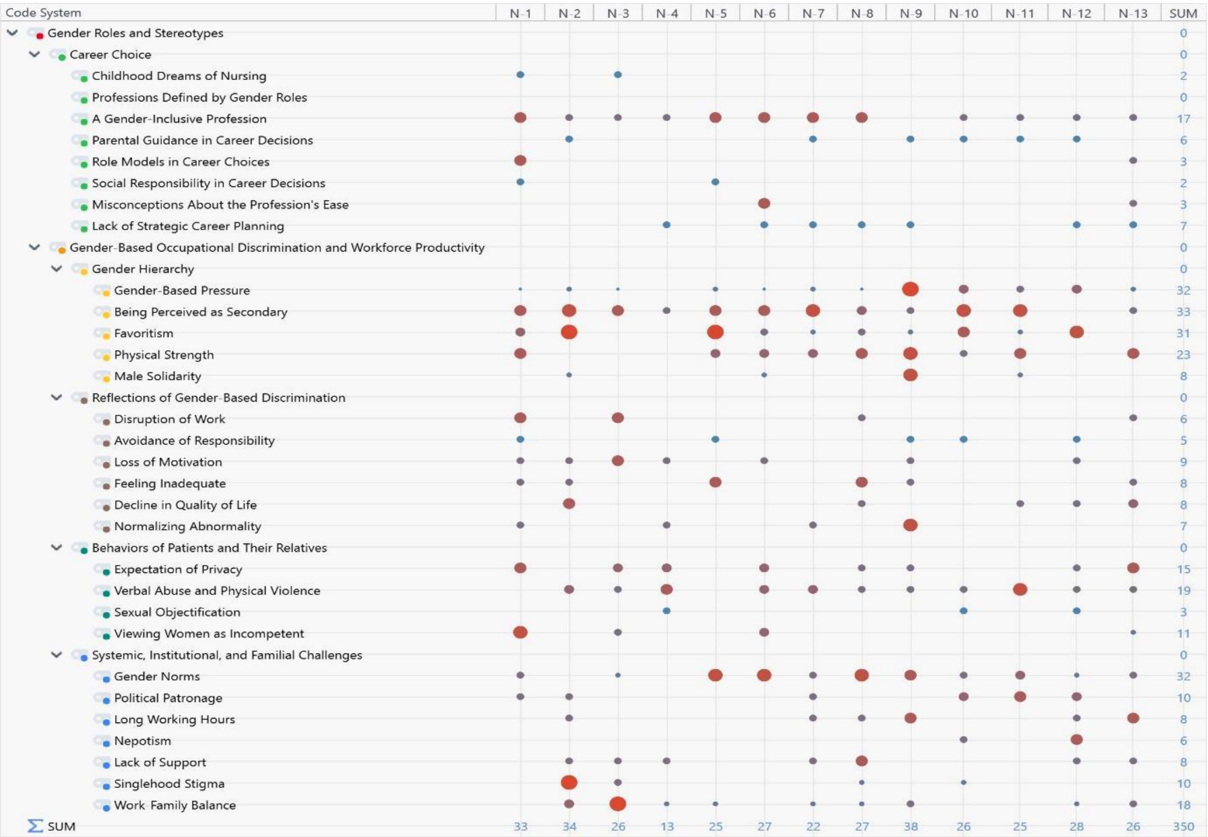

Figure 1. General view of the content analysis

The code-matrix browser generated using MAXQDA, offering an overview of the categories and codes derived from field research findings. The size of the circles in the matrix reflects the density distribution of participant statements within the coding system. For instance, statements by Participant N-2 predominantly cluster under the codes for favoritism and stigma against singlehood.

As illustrated in Figure 1, the findings are categorized under two groups explaining one main theme: "gender roles and stereotypes." Among these, the "career choice" category has no subcategories, while the second category, "gender-based occupational discrimination and workforce productivity," is divided into four subcategories. A general review of the content analysis reveals that, with the exception of the "professions defined by gender" code, all codes are represented with varying levels of intensity.

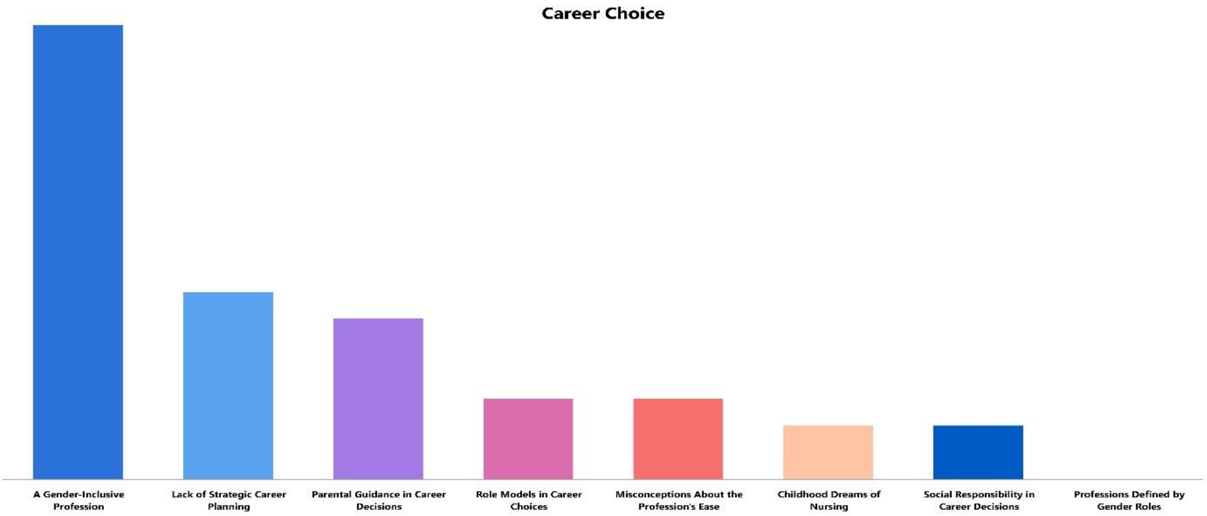

Figure 2. Career Choice in Nursing

Figure 2 displays the distribution of codes regarding participants' reasons for choosing nursing as a profession. Researchers categorized these reasons as childhood dreams, women's profession, gender-neutral profession, family guidance, role modelling, social responsibility in career choice, the perception of nursing as an easy profession, and a lack of strategic career planning. Notably, the "women's profession" code was the only one with no associated participant statements, suggesting that participants did not perceive nursing as exclusively a women's profession. The predominance of the "gender-neutral profession" code further supports this finding.

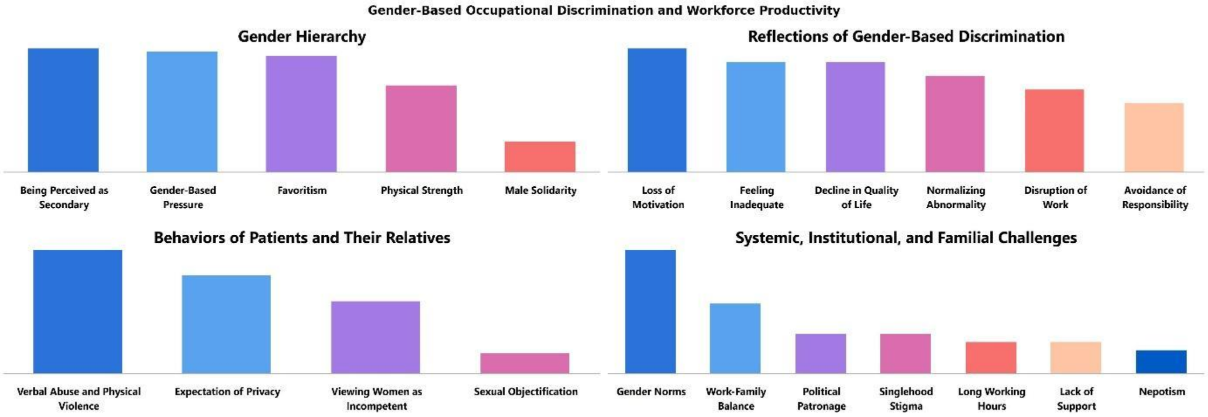

Figure 3. Gander-based occupational discrimination and workforce productivity in nursing

Figure 3 highlights the societal, institutional, and familial causes of gender-based occupational discrimination experienced by participants and its impact on nursing productivity and nurses' psychological well-being. A visible gender hierarchy, often favoring men, was frequently coded under the subcategory of "gender hierarchy." This includes findings that women are seen as less valuable than men, their opinions are less respected, and they face greater occupational pressure simply for being women. Promotion processes also tend to favor men, reinforcing this hierarchy.

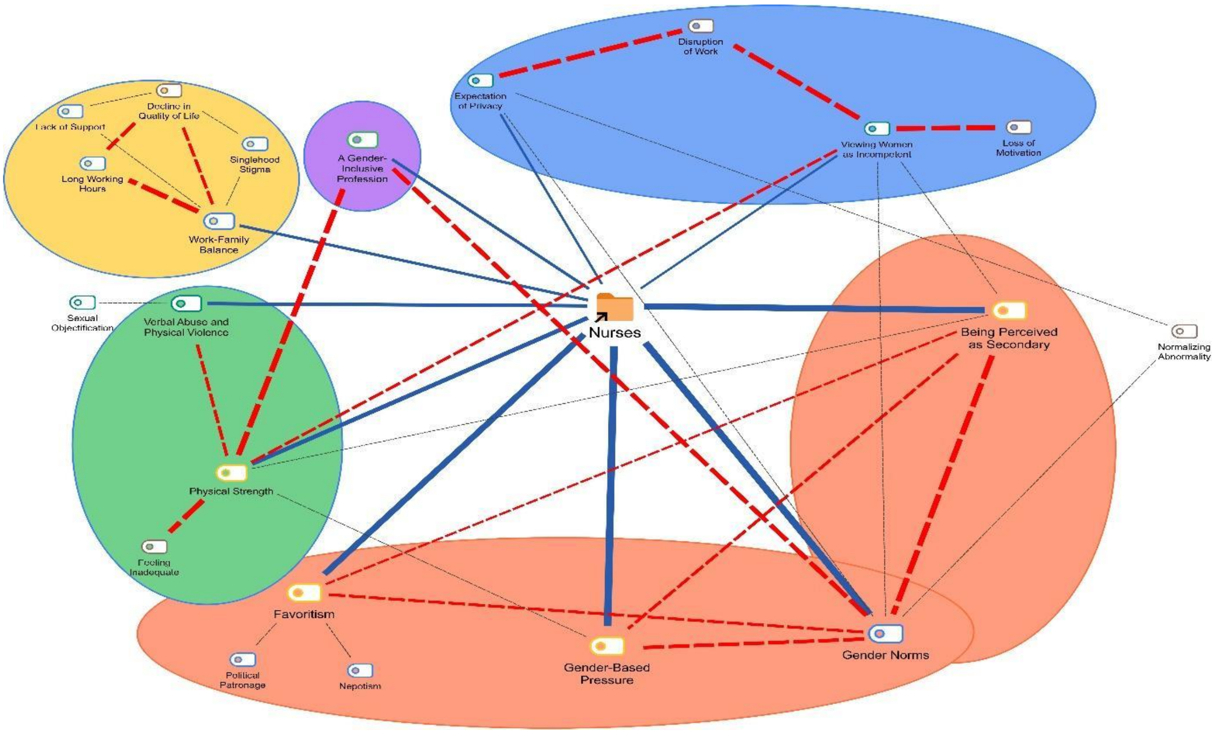

Figure 4. General view of the all cases

Figure 4 summarizes the patterns among the codes identified in all participant statements during content analysis. Starting with the "secondary status" code and moving clockwise, the blue lines represent the ten most frequently mentioned codes, while red and black lines indicate co-occurring codes, with line thickness reflecting their intensity. The dashed red lines mark regions where co-occurring codes are concentrated. The colored circular areas highlight topics emphasized in the discussion and conclusions sections of our study. Below, we present the unique discussion points and conclusions derived from our findings under each category, comparing them with similarities or contrasts in the literature.
